# Clinical assessment and pathophysiology of *Bothrops* venom-related acute kidney injury: a scoping review

**DOI:** 10.1590/1678-9199-JVATITD-2019-0076

**Published:** 2020-07-10

**Authors:** Polianna Lemos Moura Moreira Albuquerque, José Hicaro Hellano Gonçalves Lima Paiva, Alice Maria Costa Martins, Gdayllon Cavalcante Meneses, Geraldo Bezerra da Silva, Nicholas Buckley, Elizabeth De Francesco Daher

**Affiliations:** 1 University of Fortaleza (Unifor), Fortaleza, Ceará, Brazil.; 2 Toxicological Information and Assistance Center, Instituto Doutor Jose Frota Hospital, Fortaleza, Ceará, Brazil.; 3 State University of Ceará (UECE), Fortaleza, Ceará, Brazil.; 4 Graduate Program in Pharmaceutical Sciences, Federal University of Ceará (UFC), Fortaleza, Ceará, Brazil.; 5 Public Health and Medical Sciences Graduate Programs, School of Medicine, University of Fortaleza, Fortaleza, Ceará, Brazil.; 6 The University of Sydney, Sydney, New South Wales, Australia.; 7 Graduate Program in Medical Sciences, Federal University of Ceará (UFC), Fortaleza, Ceará, Brazil.

**Keywords:** Bothrops, Snakebite, Acute kidney injury (AKI), Envenoming

## Abstract

*Bothrops* are one of the most common medically important snakes found in Latin America. Its venom is predominantly hemotoxic and proteolytic, which means that local lesion (edema and redness) and hemorrhagic symptoms are recurrent in envenoming by this snake. Although hemorrhage is usually the major cause of death, snakebite-related acute kidney injury is another potentially fatal clinical complication that may lead to chronic kidney disease. The present review highlights the main studies on *Bothrops* venom-related acute kidney injury, including observational, cross-sectional, case-control and cohort human studies available up to December 2019. The following descriptors were used according to Medical Subject Headings (MeSH): on Medline/Pubmed and Google Scholar “acute kidney injury” *or* “kidney disease” *and* “*Bothrops*”; on Lilacs and SciELO “kidney disease” *or* “acute kidney injury” *and* “*Bothrops*”. Newcastle-Ottawa quality assessment scale was used to appraise the quality of the cross-sectional and cohort studies included. The selection of more severe patients who looked for health care units and tertiary centers is a risk of bias. Due to the methodological heterogeneity of the studies, a critical analysis of the results was performed based on the hypothesis that the design of the included studies influences the incidence of acute kidney injury. Fifteen human studies (total participants 4624) were included according to stablished criteria. The coagulation abnormalities (hemorrhagic symptoms, abnormal fibrinogen and activated partial thromboplastin time) were associated with acute kidney injury in the most recent studies reported. The findings observed in this review provide up-to-date evidence about the acute kidney injury pathogenesis following *Bothrops* syndrome. Studies pointed out that coagulation abnormalities comprise the major pathway for acute kidney injury development. This review may improve patient management by primary healthcare providers, allowing earlier diagnosis and treatment of *Bothrops* venom-related acute kidney injury.

## Background

Snakebite envenoming was considered again a neglected tropical disease by World Health Organization (WHO) in 2017 [1]. Renal damage is a complication in this scenario and can be associated with higher risk of death.

Acute kidney injury (AKI) defined as an increase in serum creatinine ≥ 0.3 mg/dL (26.5 µmol/L) within 48 hours or increase in ≥ 1.5 times baseline or urine volume < 0.5 mL/kg/h for 6 hours [2]. The incidence of AKI in developing countries varies between 0.7% to 31.0% and there is great heterogeneity in different areas [3]. In this context, AKI is reported in young persons caused by a single clinical condition, such as infectious diseases and envenoming [3, 4].

In Latin America, most snakebites are caused by *Bothrops* snakes and lead to hemotoxic envenoming. Snakebite-related acute kidney injury (AKI) is a common severe complication of this envenoming that may cause death [5-7]. Severe kidney injury may also require renal replacement treatment (RRT) ranging from 0.7 to 75.0% of cases to maintain the homeostasis [5, 6, 8-11]. Moreover, hemotoxic snake venoms can provoke kidney abnormalities that contribute to chronic kidney diseases in developing countries [12, 13].

Hence, this review focuses on the pathogenesis of *Bothrops* venom-related AKI, highlighting current studies under a perspective of clinical application. According to the literature, the main pathogenic mechanism of *Bothrops* venom-induced AKI is attributed to coagulation abnormalities [14]. However, direct action of venom on kidney and its hemodynamic effects, myoglobinuria, hemoglobinuria and immunologic mechanisms may play a minor role as reported in experimental studies [15-21].

## Methods

This review was developed based on the Preferred Reporting Items for Systematic Reviews and Meta-Analyses (PRISMA). Observational, cross-sectional, case-control and cohort epidemiological studies available online were selected, mandatorily those conducted in individuals ≥ 18 years old and victims of *Bothrops* bites.

The search was performed in the following databases: Medline/Pubmed, SciELO and Lilacs, including articles published until December 2019. Some alternative sources, such as reference lists from other studies and reviews on the same topic, were consulted to ensure the inclusion of relevant articles. The search was limited to English, Spanish and Portuguese.

The following descriptors were used according to Medical Subject Headings (MeSH): on Medline/Pubmed and Google Scholar “acute kidney injury” *or* “kidney disease” *and* “*Bothrops*”; on Lilacs and SciELO “kidney disease” *or* “acute kidney injury” *and* “*Bothrops*”.

The selection of the papers was performed in a standardized manner by two authors independently. Possible discrepancies were analyzed by a third author. When the title and abstract were not elucidative, a full article analysis was performed.

**Figure 1.**
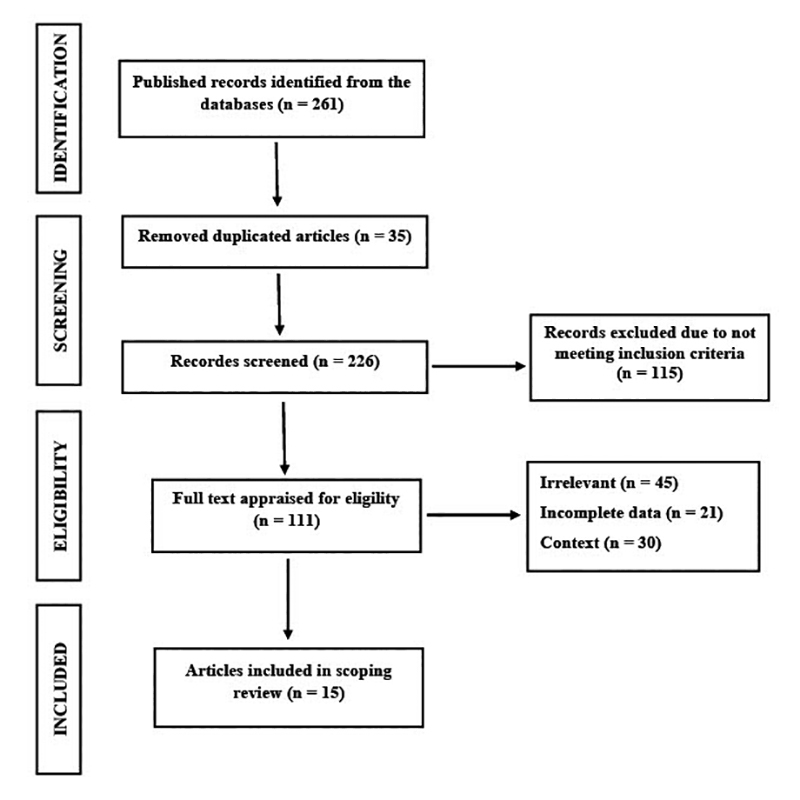
PRISMA flowchart showing the study design process.

We identified 261 articles, of which 115 were excluded from the study after reading the title and abstract. A total of 111 articles were selected for full text evaluation, and 95 studies were excluded. The final inclusion therefore comprised 15 studies that met the proposed inclusion criteria and were used for the construction of this review (Figure 1). Newcastle-Ottawa quality assessment scale (NOS) was used to appraise the quality of the cross-sectional and cohort studies included. The selection of more severe patients who looked for health care units and tertiary centers is a risk of bias. Few human studies were included according to inclusion criteria, then, some epidemiological and experimental studies were reported in this scoping review.

### ***Bothrops* snakes: variability and distribution**

The variability of snakes in *Bothrops* genera is remarkable [22]. There are more than 30 endemic species distributed from southern Mexico to Brazil [23]. The incidence and severity of snakebites depend on environmental and human factors [24, 25].

**Figure 2.**
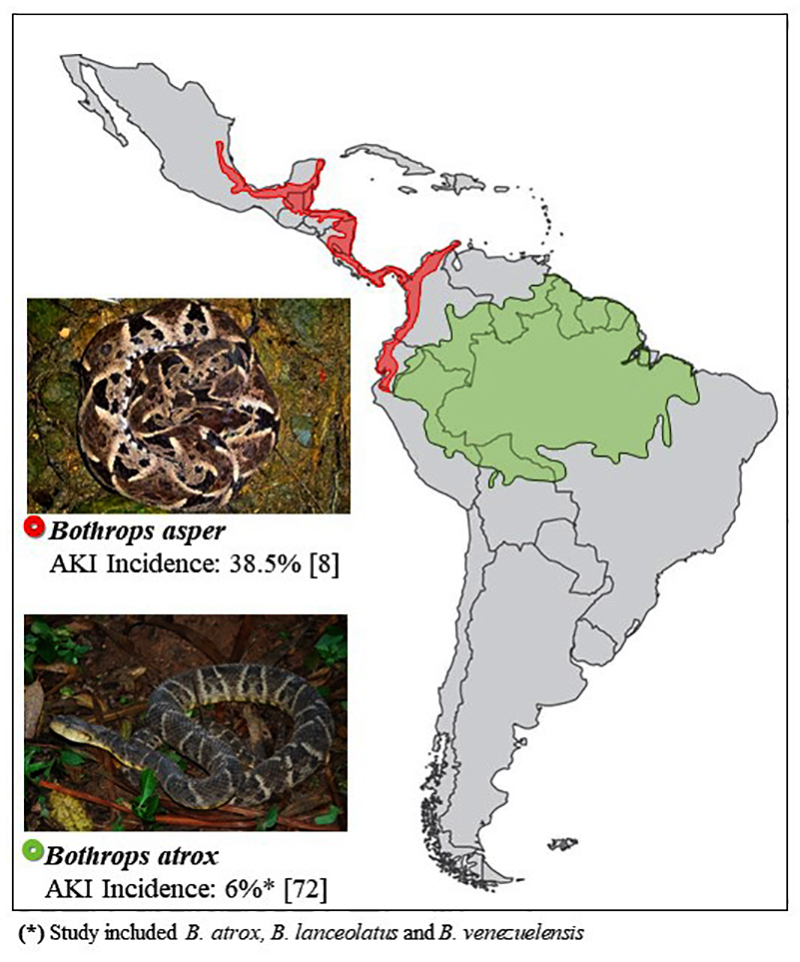
Geographical distribution of *Bothrops asper* (photo courtesy of Livia Correa, Special Laboratory of Zoological Collections, Butantan Institute, Brazil) and *Bothrops atrox* (photo courtesy of Marcelo Duarte, Laboratory of Zoological Collections, Butantan Institute, Brazil) and respective incidence of AKI in Latin America.

**Figure 3.**
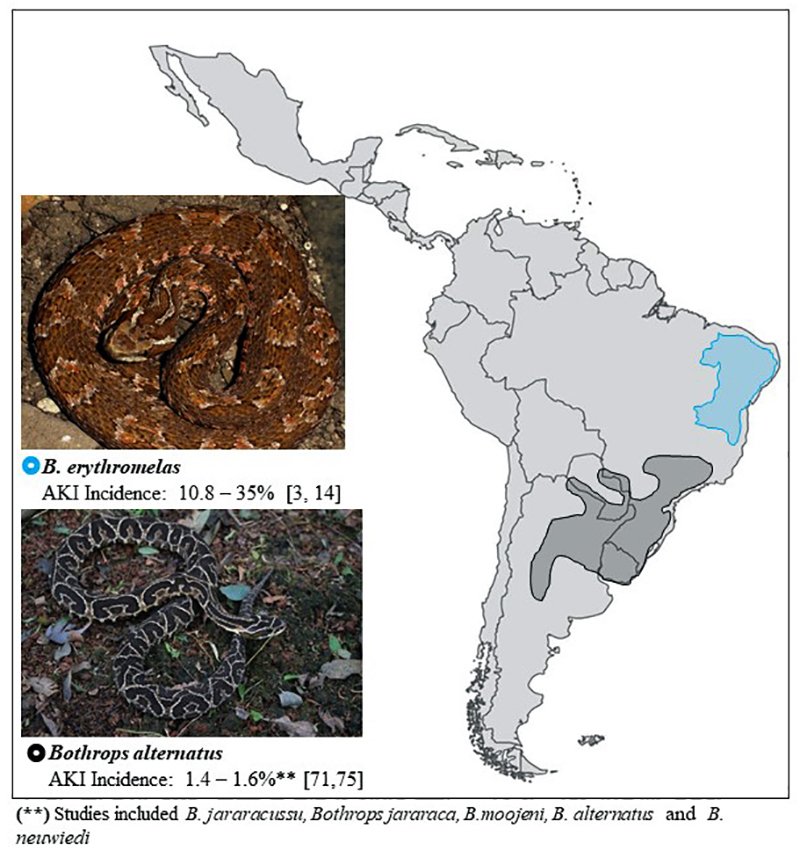
Geographical distribution of *Bothrops erythromelas* (photo courtesy of Bruno Cardi, Laboratory of Toxicology and Molecular Neuropharmacology, State University of Ceará, Brazil) and *Bothrops alternatus* (photo courtesy of Marcelo Duarte) and respective incidence of AKI.

**Figure 4.**
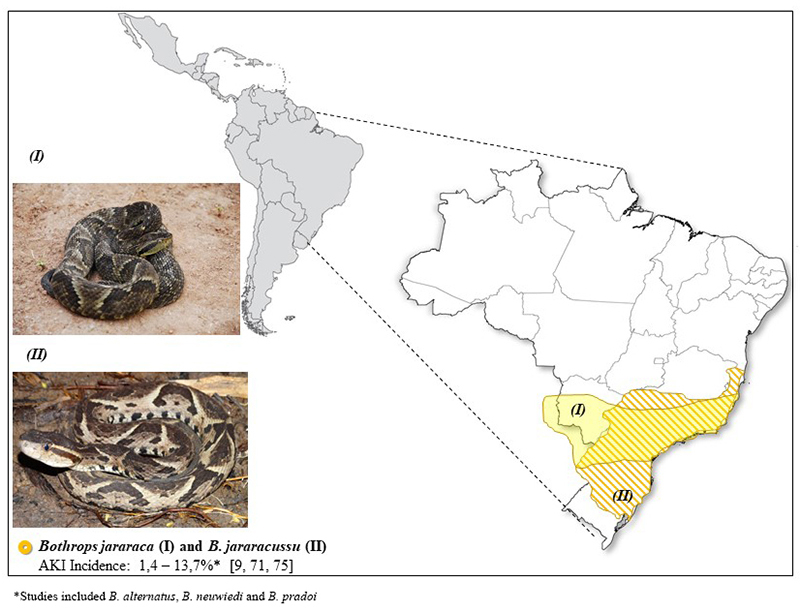
Geographical distribution of *Bothrops jararaca* (photo courtesy of Marcelo Duarte) and *Bothrops jararacussu* (photo courtesy of Paulo Bernarde, Federal University of Acre) and the incidence of AKI.

**Figure 5.**
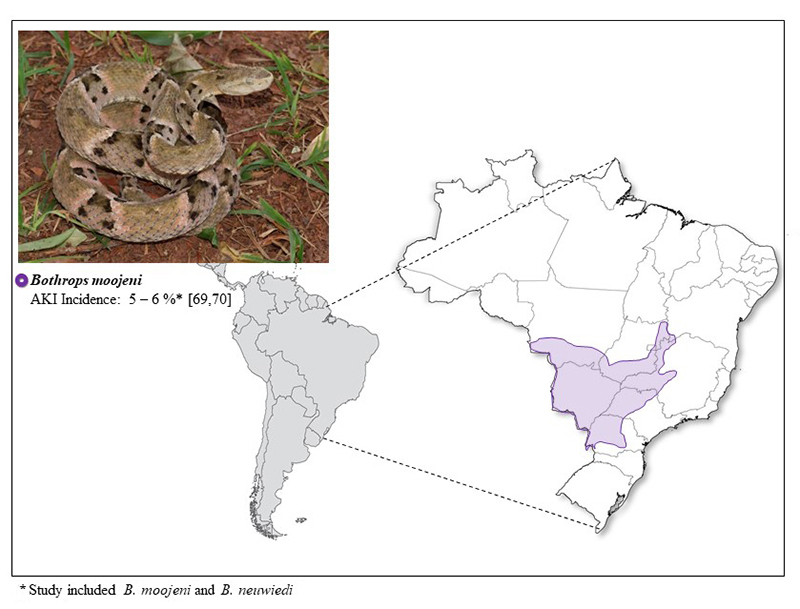
Geographical distribution of *Bothrops moojeni* (photo courtesy of Paulo Bernarde) and the incidence of AKI.

Snakebites are an occupational hazard in rural tropical areas [26-29]. These accidents are more frequent during rainy seasons, the most affected group is young men and lower limbs are the most frequently injured body parts [3]. The distribution of the main *Bothrops* species in Latin America, based on WHO, can help in the identification of snakes and in the diagnosis of envenoming [23] (Figures 2, 3, 4 and 5).

### ***Bothrops* venom**

**Figure 6.**
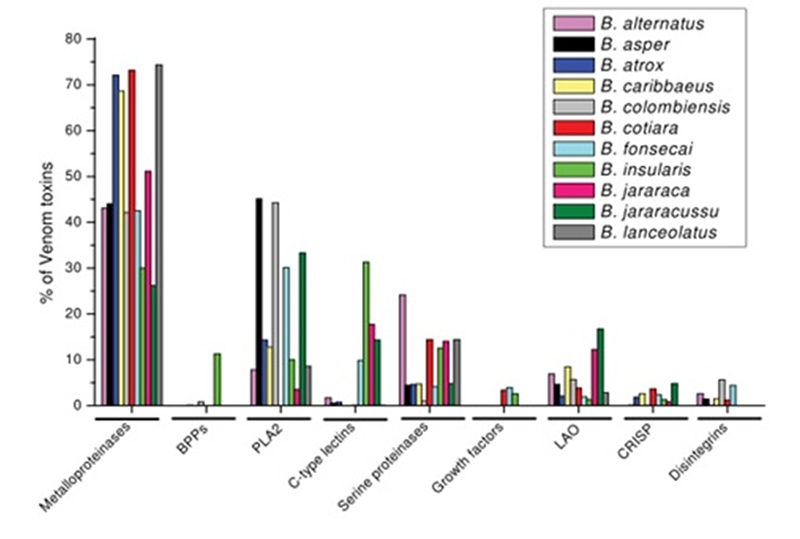
Relative abundance of the major toxin classes in bothropic venoms determined by proteomic analysis. Abundance expressed as a percentage of the total number of toxins identified in each analysis. BPPs: bradykinin-potentiating peptides; PLA_2_: phospholipase A_2_; LAO: L-amino acid oxidase; CRISP: snake venom cysteine-rich secretory proteins [39]. (Reprinted with permission from: Cardoso KC, et al. A transcriptomic analysis of gene expression in the venom gland of the snake *Bothrops alternatus* (urutu). BMC Genomics. 2010;11:605.)

The notorious variability in *Bothrops* genera contributes to the wide range of venoms and their biological effects. Several studies have compared the different characteristics among *Bothrops* venoms [15, 30-39]. There are several biochemical families of toxins in the venom of *Bothrops* species including snake venom metalloproteinases (SVMs), snake venom serine proteinases (SVSPs), L-amino acid oxidases (L-AAOs) and phospholipases A_2_ (PLA_2_s) [38] (Figure 6).

SVMs degrade all types of extracellular matrix proteins, disrupt cellular matrix and adhesion, activate chemokines and cytokines, and cleave cell surface receptors. Moreover, they induce apoptosis of vascular adhesion cells. Class P-III SVMs besides inducing hemorrhage, they activate coagulation factors, inhibit platelet aggregation, and provoke local symptoms. For example, jararhagin acts on renal toxicity [18, 38, 40-44]. PLA_2_s have a pivotal role in inflammation by activating arachidonic acid that leads to generation of eicosanoids (prostaglandins and leukotrienes). They also stimulate the hypothalamic-pituitary-adrenal axis to produce adrenocorticotropic hormone, corticosteroids, vasopressin and acute phase proteins; as well as cause local manifestations at the bite site and hemodynamic changes [43, 45-48]. SVSPs are highly expressed in kidneys. They possess thrombin-like action with fibrinolytic activity leading to blood coagulation disturbances, vasodilatation and hypotension through nitric oxide-dependent guanylyl cyclase, convert kininogen to kinin, following vascular smooth muscle relaxation, enhance tubular reabsorption of Na in the collecting duct [43, 49, 50]. L-AAOs cause endothelial injury, platelet aggregation, cellular apoptosis to DNA damage and nephrotoxicity as well as cytotoxicity in Madin-Darby canine kidney (MDCK) cells.

Snake venom contains multiple proteolytic toxins that may cause systemic as well as renal hemodynamic changes. These toxins also have thrombin-like action and fibrinolytic activity [43]. The SVMs are the most important toxins that are able to degrade all types of extracellular matrix proteins, disrupt cellular matrix and cellular adhesion, cleave cell surface receptors [43], activate chemokines and cytokines [41], as well as induce apoptosis of vascular adhesion cells [18, 40]. This toxin can activate coagulation factors, inhibit platelet aggregation and induce local symptoms at the site of the bite [42, 44].

The correlation between venoms from different species and their key toxic compounds are the mainstay of the specific treatment of *Bothrops* snakebites [30, 51, 52]. The similarities help the creation of next-generation antivenom therapies, which might improve the management of the patients [53].

### ***Bothrops* envenoming: incidence and implications**

***Clinical manifestations***

The expression “*Bothrops* syndrome” describes the variety of manifestations caused by *Bothrops* envenoming [54]. However, the ontogenetic variations in venom composition might have clinical implications [55]. The most important feature of *Bothrops* envenoming is the local effect of proteolytic toxins due to snakebite. After the bite, discrete bleeding is common at the venom inoculation site. Edema, pain, redness and bruising can also be observed [56]. Local inflammation could lead to amputation, especially in patients bitten in the fingers and among those who developed blisters and abscesses at the bite site. Systemic bleeding and renal failure are also possible outcomes [57].

*Bothrops* venom contains various biologically active peptides that may elicit an inflammatory response and contribute to cell and tissue damage as well as hemostatic abnormalities. It could activate coagulation factor X, prothrombin and lyses fibrinogen, which leads to hypofibrinogenemia and the production of fragile fibrin [6]. As a consequence, consumption coagulopathy and blood incoagulability may appear, which may lead to death [58]. Systemic effects include spontaneous hemorrhage, disseminated intravascular coagulation and cardiovascular shock secondary to hypovolemia and vasodilation [59]. Cerebral hemorrhage, following multiple manifestations of coagulopathy and AKI, is a severe manifestation and was described in the literature [60].

Coagulation disturbances, bleeding and the presence of life-threatening complications (cardiovascular shock, AKI and vital organ damage) define the systemic envenomation [61]. Besides, the severity of clinical symptoms has a strong association with the quantity of circulating venom [62]. The extension of edema on hospital admission and the presence of necrosis define the local envenoming grade and guides the number of antivenom vials that must be used in specific treatment [63].

**Mortality due to *Bothrops* envenoming**

Death following snakebites are infrequent [64]. The fatality rate due to *Bothrops* envenomation also varies among countries and regions within a country [61]. The average annual incidence in American countries is about 57,500 snake bites (6.2 per 100,000 people) and mortality is close to 370 deaths (0.04 per 100,000 people), that is, between one third and half of the previous estimates [24]. In Brazil there were 202,288 cases of *Bothrops* accidents between 2004 and 2016, and only 752 deaths, resulting in a lethality rate of 0.37% [65]. Comparative toxicological and biochemical studies performed on the venoms of adult *B. asper* specimens from Panama revealed high similar profile of activities with quantitative differences among venoms from different countries (Guatemala, Honduras, Costa Rica, Colombia, and regions of Mexico). Interestingly, the manufactured polyvalent antivenom neutralized the lethality of the venoms of the four regions and showed a pattern of strong cross-reactivity against the various proteins of these venoms [51]. In Brazil, Costa Rica and Panama, antivenoms are freely available for patients in all reference hospitals and health centers [61], which is an effective measure to decrease mortality. Moreover, studies have suggested a delayed time between snakebite and antivenom administration in patients with higher incidence of AKI, therefore it is important to observe the antivenom distribution chain [5, 66].

### ***Bothrops* venom-related AKI: burden in developing countries**

**Incidence of AKI following *Bothrops* envenoming**

There are several epidemiological studies that describe AKI. However, only a few applied the AKI definition according to KDIGO, AKIN or RIFLE criteria [5, 8, 11, 14, 56, 67-74]. Data from retrospective series described a great variable in AKI incidence, 1.4 to 44.4% [5, 8, 11, 56, 66-74]. Therefore, the real incidence of AKI due to *Bothrops* envenoming is underestimated, not only because of the ambiguity of the definition, but also the absence of notification in some rural areas [63] (Table 1).

**Table 1.** Incidence of nephrotoxic AKI after bothropic envenoming.

Definition of AKI	Species*	n	AKI n (%)	Dialysis n (%)	Coagulopathy n (%)	Mortality n (%)	Reference
**	*Bothrops* sp*.*	67	7 (10.5%)	-	-	-	[68]
-	*Bothrops moojeni*	37	0	-	27 (72.7%)	1 (2.9%)	[56]
-	*Bothrops jararaca, Bothrops jararacussu*	27	-	9 (33.3%)	-	-	[11]
-	*Bothrops* sp.	114	7 (6%)	-	16 (14%)	0	[67]
**	*Bothrops moojeni*	57	3 (6%)	-	-	-	[69]
**	*Bothrops moojeni,* and *Bothrops neuwiedi*	292	15 (5%)	-	158 (54%)	3 (1%)	[70]
-	*Bothrops jararacussu*	29	4 (13.7%)	2 (7.0%)	14 (48.2%)	3 (10%)	[9]
**	*Bothrops jararaca* (97.5%), *Bothrops jararacussu, Bothrops neuwiedi, Bothrops moojeni, Bothrops alternatus* and *Bothrops pradoi*	3,139	50 (1.6%)	22 (0.7%)	1729 (55.1%)	9 (0.29%)	[75]
-	*Bothrops lanceolatus, Bothrops venezuelensis* and *Bothrops atrox*	60	1 (6.0%)	-	33 (55%)	0	[72]
**	*Bothrops jararaca, Bothrops alternatus* and *Bothrops neuwiedi*	73***	1 (1.4%)	-	34 (60.7%)	0	[71]
-	*Bothrops asper*	39	15 (38.5%)	13 (33.3%)	31 (79.5%)	40 (10.3%)	[8]
-	*Bothrops* sp.	165	0	-	-	1 (0.5%)	[124]
RIFLE and AKIN	*Bothrops erythromelas*	276	30 (10.8%)	15 (5.43%)	97 (35.1%)	4 (1.5%)	[5]
AKIN	*Bothrops* sp.	186	24 (12.9%)	31 (16.7%)	180 (97.3%)	0	[66]
KDIGO	*Bothrops erythromelas*	63	22 (35%)	-	44 (70%)	0	[14]

*Identification of the species of *Bothrops* carried out in a few cases. **Elevated levels of serum creatinine which later returned to normal range. ***Under 15 years old. (-) Not described.

**Characteristics of *Bothrops* venom related AKI**

The acute kidney injury in *Bothrops* accidents are commonly oliguric, severe and occurs within the first hours after the snakebite [6, 10, 59, 61, 63, 75-78]. Da Silva et al. [79] described 29 patients with AKI due to bothropic and Crotalic accidents in an intensive care unit. Crotalic accidents commonly provoke oliguria or anuria later than *Bothrops* accidents. Therefore, it is important to consider the oliguria when the hydration is stablished.

### Pathophysiology

***The role of experimental studies for scientific evidence***

The direct effects of *Bothrops* venom in isolated renal perfusion experimental models have provided major insights about the pathogenesis of AKI [15, 18, 19, 21, 48, 80, 81] (Table 2). AKI could vary according to differences in venom potency and composition [31, 32]. The renal cells exposed to *Bothrops* venom mimic the changes in the human body. The concentration of venom in the perfusion fluid was estimated according to the amount inoculated by *Bothrops* snakes in a person weighting 60 kg. Decreased renal vascular resistance is observed and can occur due to blockade of either [Na^+^] and [Ca^2+^] channels or opening of [K^+^] channels [43].

**Table 2.** Renal hemodynamic changes in experimental studies with *Bothrops* venom

Species	Toxin (crude/compound)	RVR	GFR	V	FENa	FEK	References
*Bothrops jararaca*	Crude	* 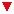	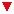	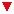	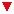	***	[80]
	Crude	** 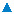	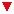	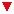	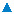	***	[81]
	Crude	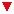	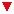	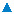	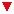	***	[84]
	Crude (southern Brazil)	−	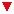	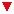	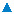	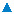	[15]
	Crude (southeastern Brazil)	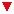	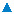	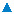	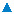	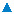	
*Bothrops moojeni*	Crude	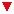	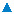	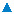	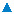	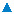	[85]
	Myotoxin I	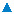	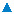	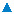	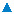	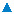	
Bothrops erythromelas****	Crude	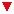	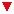 / 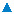	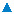	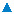	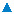	[86]
*Bothrops marajoensis*	Crude	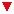	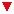	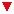	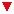	−	[48]
	PLA_2_	−	−	−	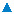	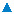	
	L-amino acid oxidase	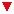	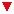	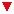	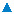	−	[19]
Bothrops leucurus*****	Crude	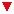	−	−	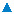	−	[21]
*Bothropoides pauloensis*	Crude	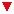	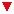	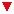	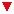	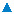	[18]

RVR: renal vascular resistance; GFR: glomerular filtration rate; V: urinary flow; FENa: fractional excretion of sodium; FEK: fractional excretion of potassium; FECl: fractional excretion of chloride. *( 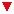
Decrease. **( 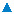 Increase. ***Not determined. ****GFR decreased at 60 min and increased at 120 min. *****The effect on urinary flow and GFR was transient and returned to normal at 120min of venom perfusion. (−) No change.

The pharmacokinetic profile in rat exposure of *Bothrops* venom revealed important aspects of the distribution and route of elimination [82, 83]. Bothropic venom may be found in renal tissue associated with morphological damage and renal dysfunction [82]. The presence of *Bothrops alternatus* venom in renal tissue was detected 30 min post-venom exposition, but it decreased progressively thereafter, in parallel with serum venom concentrations [82]. Immunohistochemistry detected it in glomeruli, proximal, distal tubules, vascular and perivascular tissue. The venom appeared in urine 3, 6- and 24-hours post-injection. Oliguria occurred from 3 hours to 7 days after venom administration [84-86], whereas proteinuria was more observed in the first 3 hours. Creatinine clearance decreased progressively until 24 to 48 hours after administration of venom, then returned to normal. Circulating venom showed biexponential kinetics, with no venom after 7 days post-exposition. Glucose, ketone, leucocyte and occult blood abnormalities occurred mainly during the first 6 hours after venom injection [82].

Moreover, systemic hemodynamic changes, such as a decrease in blood pressure occurred in many animal models after perfusion with bothropic venom [48, 80]. Vascular permeability may contribute to hypovolemia, but further studies are required [43]. Most animal toxins close calcium and sodium channels leading to hypotension. These ion channels contribute to hemodynamic effects of *Bothrops* enzymes and peptides [87].

The identification of new *Bothrops* toxins, such as vascular endothelial growth factor (VEGF), contribute to the understanding of kidney injury pathogenesis [88, 89]. A bioactive proline-rich decapeptide, part of C-type natriuretic peptide precursor from *Bothrops jararaca*, Bj-BPP-10c, displayed a strong and sustained anti-hypertensive action. The activation of arginosuccinate synthetase was the major protein linked to the peptide and led to an increase of nitric oxide [89]. The Bj-BPP-10c could be considered as a lead molecule to develop therapeutic agents for the treatment of various diseases based on NO deficiency as cause or effect. [89].

***Main pathways***

The pathways associated with AKI development in bothropic envenoming are based on scientific evidence distributed according to the main clinical manifestations in the literature.

The most important pathway in *Bothrops* venom-related acute kidney injury is probably coagulopathy. However, almost all included studies were cross-sectional and just three presented clear definition of acute kidney injury according to stages of KDIGO, AKIN or RIFLE [5, 14, 66]. These studies reported the abnormal activated partial thromboplastin time (aPTT), bleeding symptoms and abnormal LDH level which are factors associated with AKI development [5, 14, 66]. The increase of LDH levels was observed in recent case reports about thrombotic microangiopathy in *Bothrops* envenoming and expressed the high cellular turnover and necrosis following the increase of creatinine levels [90, 91].

**Figure 7.**
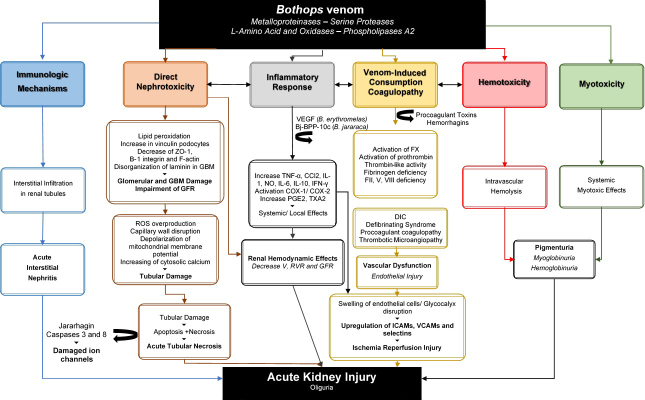
Schematic representation of pathophysiology of bothropic venom-related AKI. The main mechanisms are based on updated scientific evidences reported in the literature. AIN: acute interstitial nephritis; ATN: acute tubular necrosis; Bj-BPP-10c: bioactive proline-rich decapeptide, part of C-type, natriuretic peptide precursor from *B. jararaca*; DIC: disseminated intravascular coagulation; RVR: renal vascular resistance; GFR: glomerular filtration rate; V: urinary flow; FENa: fractional excretion of sodium; FEK: fractional excretion of potassium; F X, F VIII, F V, F II: coagulation factors X, VIII, V, II; ROS: reactive oxygen species; VEGF: vascular endothelial growth factor from *B. erythromelas*; GBM: glomerular basement membrane; ZO-1: tight junctional protein; GFB: glomerular filtration barrier; VICC: venom- induced consumption coagulopathy.

In general, the mechanisms of *Bothrops* venom-induced AKI have been attributed to [63, 92] (Figure 7):

- the direct action of venom on kidney and its hemodynamic effects;
- myoglobinuria;
- hemoglobinuria;
- glomerular microthrombi deposit due to coagulation abnormalities;
- immunologic mechanisms in a minor proportion.

***Direct nephrotoxicity***

The evidence of direct nephrotoxicity due to *Bothrops* venom comes from studies in animal models and cell cultures [15, 18, 19, 21, 48, 80, 81, 84-86, 93]. Although there were no human studies on it, the importance of the animal models offered an overview about this pathway. The hemodynamic changes in kidneys reported above (Table 2) varied according to the snake species, but most cases presented a decrease in renal vascular resistance (RVR), glomerular filtration rate (GFR) and urine flow (V), an increase of excretion of Na and K. Some venoms (*Bothrops jararacussu*, *B. erythromelas* and *B. moojeni*), however, presented an increase of GFR and V [85, 86, 93].

Renal pathologic changes in patients with AKI caused by snakebites include tubular necrosis, cortical necrosis, glomerulonephritis and vasculitis. Moreover, the tubular epithelial cells are the main targets of these venoms [43]. Glomerular changes seem to be responsible for the proteinuria and could contribute to nephrotoxicity, demonstrated for the first time in an animal model after intravenous administration of *B. moojeni* venom in rats. Mesangiolysis, microaneurysm formation and pedicel damage were consequences of the high proteolytic and PLA_2_ activities of this venom [94]. The leakage of electrolytes [Na^+^], [K^+^] in experimental models represent this tubular injury. Castro et al. [95] reported for the first time the direct toxicity of *B. jararaca* venom on isolated rat renal proximal tubules. The study demonstrated the direct effect of venom on proximal tubules independently of extracellular calcium and partially mediated by lipid peroxidation. The administration of antivenom therapy, simultaneous or up to 15 min, prevented or delayed the tubular toxicity.

Several studies have confirmed the involvement of *Bothrops* venom components in apoptosis [15, 17-19, 21, 40, 42, 46]. De Morais et al. [21] analyzed the cellular death induced by *B. leucurus* venom through flow cytometry with annexin V and propidium iodide. They concluded that death occurred predominantly by necrosis but may involve apoptosis in lower concentrations of the venom. In 2014, Collares-Buzato and Cruz-Hofling [96] reported that in renal corpuscle, the decrease in glomerular content of ZO-1, the disorganization of laminin within the glomerular basal membrane and the increase in vinculin podocytes expression led to the impairment of the glomerular filtration barrier. The cell-cell and cell-matrix adhesion proteins seem to be molecular targets in the *B. moojeni* venom-induced kidney injury [96]. Dantas et al. [19] published the renal alterations caused by *Bothrops* venom in MDCK cells. L-AAOs were cytotoxic to MDCK cells and induced late apoptosis, so these proteins acted like nephrotoxic compounds. 

Similarly, Marinho et al. [18] evaluated the effect of *B. pauloensis* venom in isolated perfused kidney and MDCK cells. They detailed the venom cytotoxicity on renal epithelial cells and apoptosis, through the caspases 3 and 7 activation, mitochondrial membrane potential collapse and reactive oxygen species (ROS) overproduction. Likewise, De Sousa et al. [17] described the apoptosis induced by *B. erythromelas* venom on MDCK cells with the involvement of the caspases 8 and 3, which probably occurs through the extrinsic pathway. The apoptosis required only low doses of venom, while necrosis required high doses and the cellular death occurred until 24 h after the exposition.

The participation of ion channels (potassium, chloride and calcium) in apoptosis requires attention [87]. The expression and activity of [Na^+^/K^+^]-ATPase associated to histological and functional alterations due to *B. alternatus* venom in rats were reported and seemed to attenuate the renal dysfunction in the early hours after envenomation [97]. The findings of proteinuria, decreased GFR, increased FE[Na^+^], increased FE[K^+^] and significantly increased gene expression of the α1 subunit of [Na^+^/K^+^]-ATPase 6 and 24 hours after venom injection were reported. Additionally, cytoskeleton alterations, F-actin disruption in Bowman’s capsule and in the brush border of renal tubules were described [97].

***Myoglobinuria***

Studies reported slight increase of creatine phosphokinase level in *Bothrops* envenoming [14, 66]. The local muscle injury caused by *Bothrops* venom could contribute to AKI. The occurrence of such muscle injury does not provoke systemic myotoxic effect, as it occurs with *Crotalus* venom [6, 98]. Melo et al. [99] reported the effects of *B. jararacussu* venom on rat and frog muscles, which confirmed the considerably increase in plasma creatine kinase (CK) activity following the injection of venom. Moura-da-Silva et al. [100] described differences of myotoxic proteins among venoms from *Bothrops* species. The myotoxicity induced by the crude venoms from *B. jararacussu*, *B. moojeni*, *B. neuwiedi* and *B. pradoi* were five to eight-fold higher than those obtained with *B. alternatus*, *B. atrox*, *B. cotiara*, *B. erythromelas* and *B. jararaca*. Nevertheless, Burdmann et al [81] carried out a study in rats exposed to *B. jararaca* venom and showed that the levels of CK were not differently affected in comparison with lactate dehydrogenase (LDH) levels.

Despite the occurrence of rhabdomyolysis induced by PLA_2_ resulting in myoglobinuria, it is not significant in *Bothrops* accidents. Nevertheless, it could contribute to AKI with hemoglobinuria. 

***Hemoglobinuria and hematuria***

Studies often reported abnormalities in urine [11]. Hematuria can be microscopic or macroscopic and the outcome is usually favorable [101]. Several mechanisms may account for the tubular injury found: mechanical obstruction by red blood cell casts, cytotoxic effects of oxidative stress induced by hemoglobin, heme iron released from red blood cells [101] and the worsening of renal vasoconstriction. Rezende et al. [80] detected hematuria and hemoglobinuria at 24 and 48 hours after intraperitoneal injection of *B. jararaca* venom in rats. Hrovat et al. [102] carried out a prospective study about renal dysfunction in dogs envenomed by cytotoxic (n = 11) and neurotoxic snakebites (n = 8), evidencing 80% of hematuria 24 hours after envenomation.

***Venom-induced consumption coagulopathy (VICC)***

This is the most likely pathway in AKI due to *Bothrops* snakes: the association between AKI and abnormal coagulation, including abnormal aPTT, hypofibrinogenemia and hemorrhagic symptoms, as soon as the increase of LDH is reported [5, 14, 66]. Snakebite induces thrombotic microangiopathy, characterized by the triad of AKI, thrombocytopenia, microangiopathic and haemolytic anaemia, which could lead to renal cortical necrosis [103-105]. Hemorrhagins contained in venoms of some snakes such as *Bothrops* spp. [40, 42, 44, 106] can cause this coagulopathy. Moura-da-Silva and Baldo [44] reported the presence of jararhagin, a metalloproteinase isolated from *B. jararaca* venom. The targets of jararhagin comprise the vascular endothelium, platelets, coagulation factors and other cell systems as inflammatory cells and their mediators [44].

Few studies reported the occurrence of thrombotic microangiopathy (TMA) in patients following *Bothrops* snakebite with thrombocytopenia, anemia with blood films showing fragmented red cells, haptoglobin consumption, increase in serum lactate dehydrogenase and a progressive increase of serum creatinine [104, 105]. Torrez et al. [107] reported the association between AKI and prolonged thrombocytopenia in a 37-year-old man admitted to a hospital after snakebite due to *Bothriopsis bilineata* in the Brazilian Amazon, presenting oliguria, dark-colored urine, incoagulability and thrombocytopenia. A study performed in adult patients after *Bothrops* snakebite revealed a strong predisposition to development of coagulation abnormalities (aPTT) and AKI development [14]. However, snakebite-associated TMA is a poorly understood phenomenon thus further studies are required to improve the management of these patients [108].

Two possible aggravating factors may contribute to the renal cortical necrosis: renal hypo-perfusion and vascular endothelial injury through a direct or indirect mechanism and the release of circulating substances (for example in intravascular hemolysis) [109]. The formation of microthrombi in blood and leukocyte migration through endothelial cells into renal interstitial compartment could contribute to vascular dysfunction. This dysfunction is an early and prominent factor in AKI, leading to ischemia/reperfusion (I/R) injury, with consequent impairment of blood flow and its regulation. In I/R injury, there is swelling of endothelial cells, disruption of the glycocalyx and endothelial monolayer, and upregulation of adhesion molecules [intercellular adhesion molecules (ICAMs), vascular cell adhesion molecule-1 (VCAMs), and selectins) resulting in an increased leukocyte-endothelium interactions with interstitial edema in the interstitial compartment [110].

***Immunologic mechanisms***

Immunologic phenomena seem to contribute to the snake venom related AKI in a minor role. There was no study included about the immunologic evidences associated with AKI development in *Bothrops* envenoming. Acute interstitial nephritis (AIN) has been observed in viperid envenoming [92, 111, 112]. The mechanism of AIN in snakebite seems to be secondary to the immunogenic effects of snake venom [113]. Severe renal failure with a prolonged clinical course is common [92] with the necessity of hemodialysis in most cases [113]. Interestingly, this clinical course was unusually prolonged when compared with tubular necrosis [112]. Low platelets and oliguric renal failure are prevalent, but urine sediment could not have anything remarkable, like eosinophiluria [112, 113].

Few cases are reporting the occurrence of AIN [111-114] and the majority of these were in Russell’s viper snake. Sitprija et al. [112] described the occurrence of AKI with prolonged oliguria in patients following Russel’s viper bite. The AIN was observed in renal biopsy revealing interstitial nephritis in addition to tubular necrosis and mesangial proliferation. No immunoglobulins and C3 were detectable in this case. Gundappa et al. [111] reported a similar case AKI following a viperid snakebite due to AIN. They described an oliguric AKI with the necessity of hemodialysis. Interestingly, the patient presented a complete recovery of renal function without the establishment of steroids. Golay et al. [114] reported five cases of AIN after Russell’s viper envenomation. These cases presented severe AKI with hemodialysis requirement. Renal biopsies showed extensive interstitial inflammation in all cases reported. Priyamvada et al. [113] carried out research aiming to detect clinical and pathological characteristics of patients who developed AIN following snakebite envenomation. Eighty-eight patients were admitted during the study period and seven biopsies were conducted. There were five patients with AIN. All these cases presented low platelets, oliguric renal failure requiring hemodialysis and received corticoids.

***Early diagnosis and management***

Investment on the training of health professionals is critical in the initial management of the patient. The use of electronic medias may improve the early diagnosis [4].

The recognition of risk factors of AKI development following *Bothrops* snakebites leads to earlier measures which reduce the renal damage [5, 6, 8, 61, 63, 70, 95]. Some information suggested a positive correlation among AKI and age, body surface area, prolonged time before treatment, bite site, hospitalization time, snake age and amount of inoculated venom. High level of lactate dehydrogenase (LDH) and local bleeding were recently independently associated with AKI development [66]. Disseminated intravascular coagulation, leukocytosis and lower serum albumin were associated with AKI in a retrospective study carried out in a region of Turkey where viper snakes are the commonest snakes [115]. It is important to look for comorbidities associated with AKI genesis, such as systemic arterial hypertension, previous diabetes, cardiac diseases or even previous kidney diseases [66].

Considering the chance of developing AKI following *Bothrops* envenoming, the immediate care must include adequate hydration and blood pressure monitoring [63, 116]. This measure could decrease the kidney damage associated with myoglobinuria, hematuria and hemoglobinuria. Moreover, it is crucial to monitor the local lesion, in order to prevent compartmental syndrome, which could lead to lactic acidosis and could worsen renal function. 

Additional tests are recommended in *Bothrops* accidents following AKI, such as laboratorial tests (coagulation tests, complete blood count, urinalysis, electrolytes, arterial or venous gasometer). Nephrologist’s opinion should be required early in cases of severe increase of creatinine (such as stages 3, 4 or 5 of KDIGO) [2].

Recently, urinary neutrophil gelatinase-associated lipocalin (NGAL) and monocyte chemoattractant protein 1 (MCP-1) were good biomarkers in predicting AKI in *Bothrops* envenoming [14]. Fractional excretion of potassium (FE[K^+^]) emerged as another diagnostic tool to predict early AKI. Positive correlation between urinary NGAL and urinary MCP-1 with proteinuria and fractional excretion of sodium (FE[Na^+^]) may indicate glomerular and tubular injury. Defects in urinary concentrations highlighted asymptomatic abnormalities, which deserve further study [14].

Studies reported chronic kidney disease, prehypertension and hypertension in snakebite patients during the follow-up period. [12]. New biomarkers could be useful to understand the continuum between AKI and CKD by the primary care physicians [13].

Proteomic and molecular approaches can be important to understand the clinical manifestations and physiopathology of victims of *Bothrops* snakebite with AKI, as well as to improve their treatment [117]. Treatment of snakebite victims must begin as soon as possible and the correct dose of antivenom is the standard treatment [63]. Developing a cheap antivenom with diminished adverse reactions is required [116]. Moreover, the presence of AKI signalizes severe envenomation and demands high doses of antivenom [118].

Recently, a significant activation of the hepatocyte growth factor (HGF)/c-met pathway in rats experimentally envenomated with *B. jararaca* venom was reported [119]. Active HGF production is enhanced in response to infectious challenges, but the increase in endogenous HGF levels is transient and insufficient. HGF targets the endothelium and epithelium of various organs to suppress local inflammation, coagulation, and apoptotic death. In various injury and disease models, HGF promotes cell survival, regeneration of tissues, and suppresses and improves chronic inflammation and fibrosis [120]. The direct anti-inflammatory action of HGF in chronic renal disease is also likely attributable to the blockade of pro-inflammatory factor nuclear kappa B (NF-κB) signaling in tubular epithelial cells [121-123].

The refractory complications associated with AKI induced by *Bothrops* envenoming should be promptly treated with RRT according to the clinical severity. The oliguria and anuria are important causes of hypervolemia, followed by hyperkaliemia in these patients, and it is the most relevant indication of RRT in *Bothrops* accidents. A recent study reported that the mechanical ventilation, hypotension and capillary leakage are independent risk factors to death in snakebite envenoming [12]. However, in the selected studies in this systematic review, none of them described the type of RRT employed.

Acute kidney injury is asymptomatic in the first stages of KDIGO. Thus, efforts to prevent, rapidly diagnose and to treat the AKI are key clinical priorities [24, 124].

## Conclusions

*Bothrops* venom related acute kidney injury is a common and potentially fatal complication of snakebite envenoming in Latin America. Scarce human studies reported the incidence of AKI and associated risk factors in this scenario. The presence of coagulopathy, such as abnormal aPTT, hypofibrinogenemia and increase of LDH pointed out the importance of this pathway in the pathogenesis of AKI development. The knowledge about the toxins in *Bothrops* venom and the experimental isolated renal models are useful tools to explain the clinical picture in humans. The pathogenesis of *Bothrops* venom related AKI includes immunologic mechanisms, coagulation disorders, pigmenturia, direct nephrotoxicity and the inflammatory response with systemic and renal hemodynamic effects. It is important to rapidly recognize *Bothrops* venom related AKI to improve treatment and reduce fatal complications. New biomarkers (NGAL and MCP-1) can be useful tools to help in early diagnosis in snakebite patients.

### Abbreviations

AIN: acute interstitial nephritis; AKI: acute kidney injury; AKIN: acute kidney injury network; aPTT: activated partial thromboplastin time; ATN: acute tubular necrosis; Bj-BPP-10c: bioactive-proline-rich decapeptide; BPP: bradykinin-potentiating peptides; CK: creatine kinase or phosphokinase; CKD: chronic kidney disease; CRISP: snake venom cysteine-rich secretory proteins; DIC: disseminated intravascular coagulation; FEK: fractional excretion of potassium; ENa: fractional excretion of sodium; GBM: glomerular basement membrane; GFB: glomerular filtration barrier; GFR: glomerular filtration rate; HGF: hepatocyte growth factor; ICAM: intercellular adhesion molecules; KDIGO: kidney disease improving global outcomes; L-AAOs: L-amino acid oxidases; LDH: lactate dehydrogenase; MCP-1: monocyte chemoattractant protein-1; MDCK: Madin-Darby canine kidney; MESH: Medical Subject Headings; NF-κB: factor nuclear kappa B; NGAL: neutrophil gelatinase- associated lipocalin; NO: nitric oxide; NOS: Newcastle-Ottawa quality assessment scale; PLA_2_s: phospholipases A_2_; RIFLE: risk, injury, failure, loss of kidney function, and end-stage kidney disease; ROS: reactive oxygen species; RRT: renal replacement treatment; RVR: renal vascular resistance; SVMs: snake venom metalloproteinases; SVSPs: snake venom serine proteinases; TMA: thrombotic microangiopathy; V: urine flow; VCAM: vascular cell adhesion molecular-1; VEGF: vascular endothelial growth factor; VICC: venom-induced consumption coagulopathy; WHO: World Health Organization; ZO-1: tight junctional protein.
